# A Review of Statistical Failure Time Models with Application of a Discrete Hazard Based Model to 1Cr1Mo-0.25V Steel for Turbine Rotors and Shafts

**DOI:** 10.3390/ma10101190

**Published:** 2017-10-17

**Authors:** Mark Evans

**Affiliations:** College of Engineering, Swansea University, Fabian Way, Crymlyn Burrows, Wales SA1 8EN, UK; m.evans@swansea.ac.uk; Tel.: +44-(0)1792295748

**Keywords:** creep, Wilshire equation, deterministic and random components, parametric and non-parametric statistical models, discrete hazard based models

## Abstract

Producing predictions of the probabilistic risks of operating materials for given lengths of time at stated operating conditions requires the assimilation of existing deterministic creep life prediction models (that only predict the average failure time) with statistical models that capture the random component of creep. To date, these approaches have rarely been combined to achieve this objective. The first half of this paper therefore provides a summary review of some statistical models to help bridge the gap between these two approaches. The second half of the paper illustrates one possible assimilation using 1Cr1Mo-0.25V steel. The Wilshire equation for creep life prediction is integrated into a discrete hazard based statistical model—the former being chosen because of its novelty and proven capability in accurately predicting average failure times and the latter being chosen because of its flexibility in modelling the failure time distribution. Using this model it was found that, for example, if this material had been in operation for around 15 years at 823 K and 130 MPa, the chances of failure in the next year is around 35%. However, if this material had been in operation for around 25 years, the chance of failure in the next year rises dramatically to around 80%.

## 1. Introduction

The prediction of long-term creep properties from short timescale experiments is rated as the most important challenge to the UK Energy Sector in a recent UK Energy Materials Review [1]. Creep strain (ε) is a function not only of stress (*τ*) and absolute temperature (*T*), but also of time (*t*)

*ε* = f_1_(*τ*, *T*, *t*) (1a)

After an initial strain on loading, a decaying creep rate ($\dot{\varepsilon }$) during the primary stage of creep is followed by an accelerating strain during the tertiary stage. A minimum creep rate (${\dot{\varepsilon }}_{m}$) occurs at the boundary of these two stages. As such, Equation (1a) is often represented in differential form

(1b) $$\dot{\varepsilon }\;=\;f_{2}(\tau ,\;T,\;t)$$

When it comes to extrapolating from short term accelerated test data, three very broad approaches can be identified. Whole creep curve methods work by relating the whole creep curve to the test conditions under which that creep curve was obtained. A single creep curve at steady uniaxial stress *τ* and absolute temperature *T* can be modelled using a general functional form

(2a) $$\varepsilon =\eta (t{,\Psi }_{1}{,\Psi }_{2},....{,\Psi }_{j},....{,\Psi }_{q})$$

where η is some non-linear function and the Ψ*_j_* are numerical parameters. At any one test condition, the Ψ*_j_* parameters are constant, but they do vary systematically with the test conditions. It is this fact that enables creep curve predictions to be made

(2b) $$\Psi_{j}=g_{j}(\tau ,T{,b}_{j,1}{,b}_{j,2},\ldots ,\;b_{j,k},....,\;b_{j,p})$$

where g*_j_* are non-linear functions, and b*_j_*_,*k*_ are additional numerical parameters that can be estimated using a suitable estimation technique. However, the form of the η and g*_j_* functions are not known and consequently the literature contains many representations of these including, for example, the Theta methodology proposed by Evans and Wilshire [2]

(3a) $$\varepsilon =\Psi_{1}(1-e^{-\Psi_{2}t})+\Psi_{3}{(e}^{\Psi_{4}t}-1)$$

with

(3b) $$\Psi_{j}=b_{j,1},1+b_{j,2}\tau +b_{j,3}T+\;b_{j,4}\tau T$$

Whilst Evans [3] derived Equation (3a) from creep deformation mechanism theory, the all important extrapolation function given by Equation (3b) is mainly empirical in nature. There are many other approaches in the literature including those by McVetty [4], Garofalo [5], Ion et al. [6], Prager [7], Othman and Hayhurst [8], Kachanov [9] and Rabotnov [10].

Secondly, parametric techniques work by relating a measured point on the creep curve to the test conditions under which that measurement was made. This point is typically the minimum creep rate or the time to rupture. Around the minimum creep rate there remains a considerable period of time where $\dot{\varepsilon }$ remains more or less constant, so that Equation (1b) reduces to

(4a) $${\dot{\varepsilon }}_{m}\;=\;f_{3}(\tau ,\;T)$$

with, following Monkman and Grant [11],

(4b) $$1/t\;\propto {\dot{\varepsilon }}_{m}$$

where here *t* represent the time at which failure occur. Because the functional form of f_3_ is not known the literature again contains many different representations of Equations (4a) and (4b). For example, Dorn [12] and Larson and Miller [13], both assumed that at a constant stress

(5a) $${\dot{\varepsilon }}_{m}=C_{0}\;\exp\;\left[-\frac{\;Q_{c}}{R^{*}T}\right]$$

where Q_c_ is the activation energy in J/mol. These two approaches then diverge with the incorporation of stress with Dorn suggesting the parameter C_0_ varies with stress, whilst the Larson and Miller model has Q_c_ varying with stress—but in both cases the form of the stress function was empirically specified (typically involving the use of polynomials in stress or the log of stress).

The literature contains many other variations including Manson and Haferd [14], Manson and Muraldihan [15], Manson and Brown [16] and Trunin et al. [17]. Unfortunately, all these parametric models suffer from parameter instability with respect to stress and temperature making reliable long term life predictions from accelerated short term testing impossible—as empirically illustrated by Abdallah et al. [18]. These empirical models are now quite old and, despite their known short comings, are still extensively used for safe life estimation. The hyperbolic tangent method [19] and the Wilshire Equation [20] can be seen as the most recent types of parametric model, with the later having the form

(5b) $$\ln[t]\;=b_{0}+b_{1}\tau^{*}+Q_{c}\frac{1000}{RT}$$

where R is the universal gas constant, τ* = ln(−ln(*τ*/*τ*_TS_)) with *τ*_TS_ being the tensile strength. Unlike the above models, a raft of recent publications [21,22,23,24,25,26] on a wide range of high temperature materials have demonstrated the parameters of this model (b_0_, b_1_, and Q_c_) are stable and so reliable long term predictions have been made from this model using short term data of no more than 5000 h duration.

Finally, there are computational/numerical approaches that often incorporate detailed deformation mechanism into finite element code to obtain creep property predictions. Many of these numerical models are based on remaining life assessment with abridged accelerated testing. In some of these approaches, for example [27,28], uniaxial test specimens are cut from removed components that have been in service for over a long time (typically over 100,000 h) and re-tested under laboratory conditions for short times until failure occurs by accelerating the temperatures (but using the in service stress). Such testing yields the remaining life or useable residual life (around 60% of remaining life). The above parametric models are then used to predict/extrapolate these residual lives to operating temperatures. Often numerical models are used to extrapolate such abridged short-term testing. An alternative to this destructive approach is the non-destructive disc test, where small discs are taken from in service components without destroying their integrity. Again numerical models can be built for this disc test, e.g., Evans [29] or parametric procedures can be used for extrapolative purposes.

What all the above studies have in common however is that they are all deterministic in nature. As the level of stress increases the time to failure diminishes and the primary component of the creep curve becomes less pronounced. These variations in creep properties are governed by (as yet not fully understood) physical laws that can be used to determine creep properties at any test condition. These physical laws are embedded into the above mechanistic models that can then be used to explain variations in creep properties as a function of test conditions alone.

**Figure 1:**
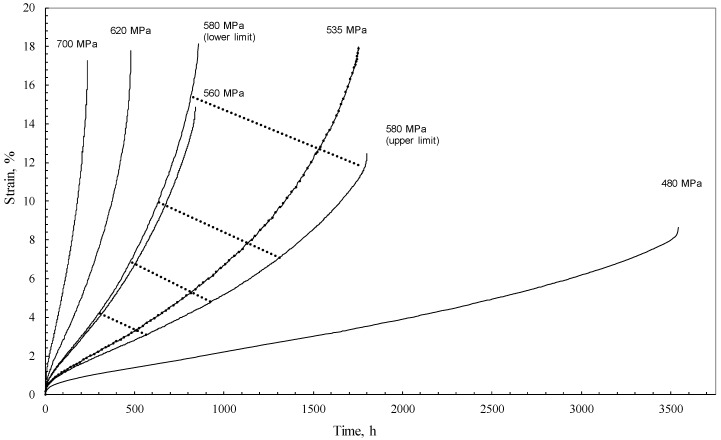
Uniaxial creep curves at 773 K for Ti-6.2.4.6 (including the band of creep curves obtained at a repeat stress of 580 MPa bounded by the maximum and minimum rupture times).

However, creep is not just a deterministic process that is predetermined by physical laws. It is also a random process. If the time function, f_1_, could be quantified, it could then be used to predict the strain at any time. Unfortunately, the nature of creep is such that this function could not then be used to predict the strain for another specimen tested under exactly the same conditions. This random component of creep is in turn very large. For example, in the NIMS [30] 1Cr-1Mo-0.25V steel database used in this paper, the time to failure at 773 K and 373 MPa varies between the limits of 125 h and 1360 h depending on the batch. A lot of the random variation seen in creep databases of this nature are down to variations in chemical composition and heat treatments experienced by the different batches of the test material. However, even when such factors are removed, failure times are still highly stochastic in nature. This was illustrated by Evans [31], who tested 15 specimens of Ti-6.2.4.6 at different temperatures and stresses. These test specimens were all cut from the same batch and tested within the same laboratory on the same make of calibrated uniaxial test apparatus. The results are reproduced in Figure 1 where it can be seen that the random variation is great enough to encompass variations induced by changes in stress.

This random component of creep is not just important because it is large in size, but also because if predictions are to be made for more than just the average time at failure, then this random component must be modelled with the same degree of vigour as displayed by all the above deterministic models. However, they are not. We can summarise the above as stating creep life has both a deterministic and a random component

(6) $${\dot{\varepsilon }}_{m}\;=\;f_{3}(\tau ,\;T)e^{f_{4}(u)}$$

where u is the random component and f_4_ is some function that describes how the random component is distributed.

The first aim of this paper is to address this shortcoming by providing a detailed, although by no means complete (as this is a very large subject area), review of statistical failure time models that describe different ways that f_4_(u) can be specified, so as to provide materials scientists with a framework for further developing their deterministic creep models, such as the Wilshire Equation, so that they become cable of providing predictions that have levels of confidence attached to them. It should be noted that these statistical models say nothing about the deterministic models reviewed above and do not imply that one model is any better at prediction than another. However, when a model of the random component is combined with the above deterministic components, they become more capable of predicting both the systematic variation with test conditions and the observed random variation at each test condition. Without this, the deterministic models can do no more than accurately predict the average safe life and not the safe life corresponding to say a 1% chance of failure. This review is provided in Section 3 of this paper and where appropriate illustrated using data on 1Cr-1Mo-0.25V steel.

The second aim of this paper is to illustrate one of the many ways that these deterministic and random component models can be combined. It is impossible in one paper to do an illustration for all of the above deterministic models reviewed above, and so the Wilshire model is selected for this purpose. This approach is selected because it has been shown to outperform the others in terms of accurately predicting the average time to failure beyond 100,000 h using very short term data (less than 5000 h). The Wilshire equation is combined with a discrete hazard based model for the random component. A hazard based model was chosen because it offers extra flexibility on distributional shape and form compared to other approaches as discussed in the review section below. Further, a discrete version of the hazard function is used because it helps empirically quantify the form of the hazard function (and because it has never been used within the context of creep failure before—whereas other approaches have [32]).

The review and illustration of combining deterministic and random creep models illustrated using the NIMS database on 1Cr-1Mo-0.25V steel [30]. This is carried out in Section 4, and conclusions are then drawn in Section 5.

## 2. The Data

This present study features forged 1Cr-1Mo-0.25V steel for turbine rotors and shafts. For multiple batches of this bainitic product, both the creep and creep fracture properties have been documented comprehensively by the National Institute for Materials Science (NIMS), Japan [30]. NIMS creep data sheet No. 9B includes information on nine batches of as tempered 1Cr-1Mo-0.25V steel. Each batch of material had both a different chemical composition and a different thermal and processing history—details of which can be found in creep data sheet No. 9B. Specimens for the tensile and creep rupture tests were taken radially from the ring shaped samples which were removed from the turbine rotors. Each test specimen had a diameter of 10 mm with a gauge length of 50 mm. These specimens were tested at constant load over a wide range of conditions: 47–333 MPa and 723–923 K. In addition to failure time (*t*) measurements, values of the 0.2% proof stress (*τ*_Y_) and the ultimate tensile strength (*τ*_TS_) determined from high strain rate (~10^−3^ s^−1^) tensile tests carried out at the creep temperatures for each batch of steel investigated were also reported.

The review section below (Section 3) is illustrated using all batches of data, whilst the discrete hazard based model outlined in Section 4 is illustrated using only a single batch of materials from this database—VaA.

## 3. Illustrated Review of Approaches to Modelling the Stochastic Nature of Creep Failure 

### 3.1. A Statistical Description of Continuous Failure at Fixed Test Conditions

Due to batch to batch variations in chemistry and heat treatment and within batch variations in microstructure, creep failure times for a high temperature material (or indeed any given material), even under fixed test conditions, are stochastic in nature. Therefor such failure times need to be described through a random variable *T*. In reality, *T* can take on a large and continuous number of different values at a given test condition, *t*_1_, *t*_2_, …, *t*_n_, with 0 ≤ *t*_1_ ≤ *t*_2_ ≤ … ≤ *t*_n_. As such it cannot be known with certainty when failure will occur and so failure must be expressed using the survivor function which gives the probability of surviving beyond a certain length of time, S(*t*)

(7a) Pr(T>t)=S(t)

The probability of failing at or before a given length of time is then given by F(*t*) = 1 − S(*t*). The probability of failure in a very small increment of time, ∆*t*, is then f(*t*) = ∆F(*t*)/∆t. F(*t*) is often referred to as the cumulative distribution function (cdf) and its derivative, f(*t*), the probability density function (pdf).

The probability of failure can also be expressed through the hazard function. This function gives the rate of failure at time *t*, given the specimen survives up to time *t*

(7b) $$h(t)=\underset{\Delta t\to 0}{\lim}\frac{\mathrm{Pr}\left(t\leq T<t+\Delta t|T\geq t\right)}{\Delta t}$$

where |*T* ≥ *t* reads given that T is greater than or equal to *t*. As such, the hazard rate is a conditional probability of failure. A conditional probability is defined as P(A|B) = P(A and B)/P(B), where in terms of the hazard rate event B is the probability of surviving a length of time t and so equals S(*t*). Event A and B would then be the probability of failing in the small increment of time ∆*t* beyond *t*, which is the pdf at time *t*. Thus

(7c) $$h(t)=\underset{\Delta t\to 0}{\lim}\frac{\mathrm{Pr}\left(t\leq T<t+\Delta t|T\geq t\right)}{\Delta t}=\frac{f(t)}{S(t)}$$

If follows from these definitions that the hazard function can also be found from the survivor function using

(7d) $$h(t)=\frac{-\mathrm{dln}[S(t)]}{dt}$$

and the cumulative (or integral) hazard function is given by

(7e) $$\Lambda (t)=\int_{0}^{t}h(t)dt=-\ln[S(t)]$$

Approaches to estimating the survivor function generally fall under three headings: parametric, non-parametric and semi-parametric. The assumption behind the parametric approach is that the form of the survivor function can be captured through a small number of parameters. For example, if failure times at a fixed test condition are normally distributed, then the survivor function is fully defined through two parameters—the mean and the standard deviation. In contrast, the non-parametric approach is model (parameter) free and as such makes no assumptions about how failure times are distributed. The semi-parametric approach combines these two approaches, for example, by specifying a base line hazard function at a particular test condition non-parametrically and then using a few parameters to model how this baseline function changes with the test conditions.

#### 3.1.1. Non-Parametric Estimation

The starting point for many non-parametric techniques is to partition time into *j* = 1 to *k* equal intervals, with k being as large as practically possible. If *n* equals the number of specimens placed on test at the same test condition and *d_j_* the number of specimens failing during the *k*th interval, then Kaplan and Meier [33] proposed the following estimator of the survivor function (for uncensored data) that has as its basis the binomial distribution

(8a) $${\hat{S}}_{km}(t_{i})=1-\frac{\mathrm{Number}\;\mathrm{of}\;\mathrm{failures}\;\mathrm{up}\;\mathrm{to}\;\mathrm{time}\;t_{i}}{n}=1-\frac{\sum_{j=1}^{k}d_{j}}{n}$$

where *d_j_* is the number of failures in time interval *j*. This estimator is also referred to as the product-limit estimator as originally these authors justified this estimator based on its properties when k tended to infinity or as the time interval tended to zero.

Nelson [34] and Aalen [35] proposed the following non-parametric estimator of the cumulative hazard function

(8b) $$\hat{\Lambda }(t_{i})=\sum_{j=1}^{k}\frac{d_{j}}{r_{j}}$$

where *r_j_* is the total number of specimens at risk (or not yet failed) just prior to time *t_i_*. The Fleming-Harrington [36] estimator of the survivor function is, from Equations (7e) and (8b),

(8c) $${\hat{S}}_{fh}(t_{i})=\exp(-\hat{\Lambda }(t_{i}))$$

The above are of course estimates (designated by the hat symbol) of the survivor function computed in the above ways, but from a population or very large sample. The standard deviation of the above estimators provides a way to quantify the possible size of the difference between the true or population survivor function—S(*t*)—and that calculated from a small sample or a randomly selected sub set of the population. The standard deviation of these estimators are in turn estimated by

(8d) $${\hat{\sigma }}_{S}=\sqrt{[1-{\hat{S}}_{km}(t_{i})]{\hat{S}}_{km}(t_{i})/n}\;\mathrm{and}\;{\hat{\sigma }}_{\Lambda }=\sqrt{\sum_{j=1}^{k}\frac{(r_{j}-d_{j})d_{j}}{{\left(r_{j}-1\right)}^{2}}}$$

In large samples, these estimates are unbiased and the Nelson Aalen estimator is then also approximately normally distributed.

**Figure 2:**
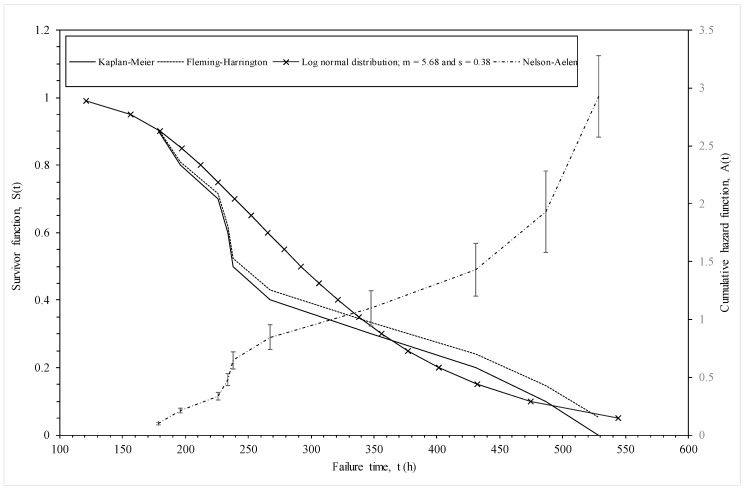
Various non-parametric and parametric estimates of the survivor and hazard functions for batches of 1Cr-1Mo-0.25V steel tested at 823 K and 294 MPa.

As an illustration, Figure 2 compares these non-parametric estimators of the survivor function for the 1Cr-1Mo-0.25V specimens in the NIMS dataset tested at 823 K and 294 MPa. At low times to failure the above two estimators provide very similar values for the survivor function, but these estimators start to diverge at around 250 h—with the Fleming-Harrington estimator exceeding the Kaplan-Meier estimator. The Nelson-Aalen estimator of the cumulative hazard function is shown on the right hand side vertical axis. The errors bars associated with the estimated cumulative hazard function, which are made equal to one standard deviation, are also shown. As can be seen, the standard error increases quite dramatically with the time to failure, making the estimates at high survival probabilities quite unreliable in a sample this small.

#### 3.1.2. Parametric Estimation

Any distribution defined for *t* ∈ (0, ∞) can be used to specify parametric survivor and hazard rate functions. A good transformation for visualising many commonly used parametric distributions is the log transformation of failure time, *Y* = ln(*T*), with *y* ∈ (−∞, ∞). Then, a whole family of distributions for Y opens up by introducing location (via parameter μ) and scale (via parameter b) changes of the form

ln(*T*) = *Y* = μ + b*Z* (9a)

where, like *T* and *Y*, *Z* is a random (but standardised) variable, *z* ∈ (−∞, ∞). To prevent the occurrence of a degenerate distribution for large values of *k*_1_ and/or *k*_2_, the following re-parameterisation is used

ln(*T*) = *Y* = μ + (b/δ)*W* = μ + σ*W* (9b)

where $\delta \;=\sqrt{\left(k_{1}k_{2}/(k_{1}+k_{2}\right)}$, σ = b/δ and where *W* is therefore another standardised random variable defined as *W* = δ*Z*.

By specifying a very general distribution for *Z*, it is possible to identify many of the familiar failure time distributions used in failure time analysis. Prentice [37] and Kalbfleisch and Prentice [38] for example defined the probability density function for *Z* as

(9c) $$f(z)=\frac{{\left(k_{1}/k_{2}\right)}^{k_{1}}}{\left\{\Gamma (k_{1})\Gamma (k_{2})\right\}/\Gamma (k_{1}+k_{2})}\frac{\exp\;\left(k_{1}z\right)\;}{{\left(1+\left\{k_{1}/k_{2}\right\}e^{z}\right)}^{\left(k_{1}+k_{2}\right)}}$$

where *Z* is said to be distributed as the logarithm of an F random variable with 2*k*_1_ and 2*k*_2_ degrees of freedom. T is described as following a four parameter generalised F distribution, T ~ GENF(μ, σ, *k*_1_, *k*_2_). Г(*k*) is the gamma function at *k*. The Appendix A to this paper also shows that the pdf of this generalised gamma distribution can be re-parameterised as a function of time

(9d) $$f(t)=\frac{{\left(k_{1}/k_{2}\right)}^{k_{1}}\lambda \;\beta }{\left\{\Gamma (k_{1})\Gamma (k_{2})\right\}/\Gamma (k_{1}+k_{2})}\frac{{\left(\lambda t\right)}^{\beta \;k_{1}-1}}{{\left(1+\{k_{1}/k_{2}\}{\left(\lambda t\right)}^{k_{1}\beta }\right)}^{\left(k_{1}+k_{2}\right)}}$$

where λ = exp(−μ) and β = 1/(δσ) = 1/b. Except, under some restricted values for *k*_1_ and *k*_2_, there is no closed form expression for the survivor and hazard functions, but they are related to the incomplete beta function and Appendix A shows how this can be computed using percentiles from the F distribution. Equation (9d) is however degenerate when *k*_1_ = *k*_2_ = ∞ and then a different specification of the pdf must be used (see Appendix A).

**Figure 3:**
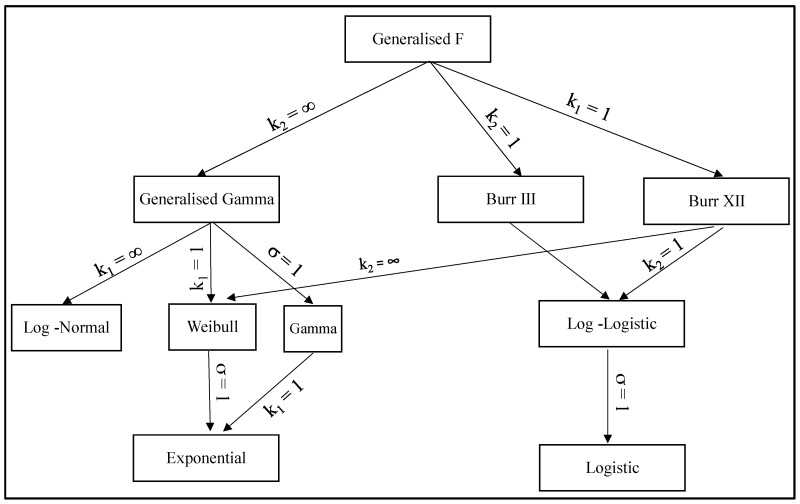
Members of the Generalised F distribution.

Particular values for these parameters define important sub families within the GENF family and these sub families are summarised in Figure 3. It can be seen that some of these distributions are commonly used within engineering. When *k*_2_ = ∞, failure times have a Generalised Gamma distribution, T ~ GENG(μ, σ, *k*_1_). There are then three well known two parameter distributions within this Generalised Gamma family. T is gamma distributed, T ~ GAM(μ, σ, *k*_1_), when *k*_2_ = ∞ and σ = 1. T is log normally distributed, T ~ LOGNOR(μ, σ), when *k*_2_ = *k*_1_ = ∞; and T is Weibull distributed, T ~ WEIB(μ, σ), when *k*_2_ = ∞ and *k*_1_ = 1. In turn, the Weibull distribution collapses to the exponential distribution when *k*_2_ = ∞, *k*_1_ = 1 and σ = 1. The family, T ~ BURR(μ, σ, *k*_1_), is obtained when either *k*_1_ = 1 (Burr III) or *k*_2_ = 1 (Burr XII). Then when *k*_2_ = *k*_1_ = 1, T has a log-logistic distribution, T ~ LOGLOGIS(μ, σ), and when *k*_2_ = *k*_1_ = 1 = σ the log-logistic distribution collapses to the logistic distribution, T ~ LOGIS(μ). The form and characteristics of all these special cases are further described in the Appendix A.

Evans [32] has shown how the parameters of these distributions can be estimated using maximum likelihood procedures. An alternative semi-parametric approach is to use the least-square procedure in conjunction with a probability plot. The procedure here is to linearise a plot of *t* against S(*t*) by finding suitable transformations of S(*t*) and possibly *t*. A least squares best fit line to the data on such a plot then yields estimates of the parameters μ (given by the intercept of the best fit line) and σ (the slope of the best fit line). However, as seen in Figure 1, the non-parametric estimate ${\hat{S}}_{km}(t_{i})$ is a step function increasing by an amount 1/*n* at each recoded failure time. Plotting at the bottom (top) of the steps would lead to the best fit line being above (below) the plotted points and so lead to a bias in the resulting parameter estimates. A reasonable compromise plotting position is the mid-point of the jump

(10a) $$\frac{1}{2}[{\hat{S}}_{km}(t_{i})+{\hat{S}}_{km}(t_{i+1})]=\frac{i-0.5}{n}={\hat{p}}_{i}$$

where *i* indexes the ordered failure times (*i* = 1 for the smallest failure time, *i* = 2 for the next smallest all the way up to n for the largest failure time), with t_1_ being the smallest failure time up to *t_n_* the largest failure time. From the Appendix A to this paper, the log of the *p*th percentile for *t* is given by

ln(*t_p_*) = μ + (b/δ){*w_k_*_1,*k*2,*p*_} = μ + σ{*w_k_*_1,*k*2,*p*_} (10b)

where w*_k_*_1,*k*2,*p*_ is the *p*th quantile of an F distribution with (2*k*_1_, 2*k*_2_) degrees of freedom. Percentiles of the F distribution are tabulated at the back of many well know engineering statistical text books (it can also be found in Excel using the FINV function). Using ${\hat{p}}_{i}$ in Equation (4a) for p in Equation (10b) allows values for w*_k_*_1,*k*2,*p*_ to be computed. Thus, when $w_{k1,k2,{\hat{p}}_{i}}$ is plotted against the ordered values for ln(*t*), ln(*t_i_*), the data points will reveal scatter around a linear line provided the data have a generalised F distribution with given values for *k*_1_ and *k*_2_.

The generality of Equation (10b) is clearly seen by considering the special case of *k*_2_ = ∞ and *k*_1_ = 1, which is the Weibull distribution, whose survivor function is shown in the Appendix A of this paper to be

(11a) $$S(t)=\exp\left[-{\left(\lambda t\right)}^{\beta }\right]$$

This can be linearised as

(11b) $$\ln(t)=-\ln[\lambda ]+\frac{1}{\beta }\ln\{-\ln[S(t)]\}=\mu +\sigma \;\ln\{-\ln[S(t)]\}$$

Then, replacing S(*t*) with the parametric estimator ${\hat{p}}_{i}$ and *t* by its ordered value *t_i_* gives

(11c) $$\ln(t_{i})=-\ln[\lambda ]+\frac{1}{\beta }\ln\{-\ln[{\hat{p}}_{i}]\}=\mu +\sigma \;\ln\{-\ln[{\hat{p}}_{i}]\}$$

Equations (11c) and (10b) imply that *w_k_*_1,*k*2,*p*_ collapses to ln{−ln[S(*t*)]} when *k*_2_ = ∞ and *k*_1_ = 1. 

**Figure 4:**
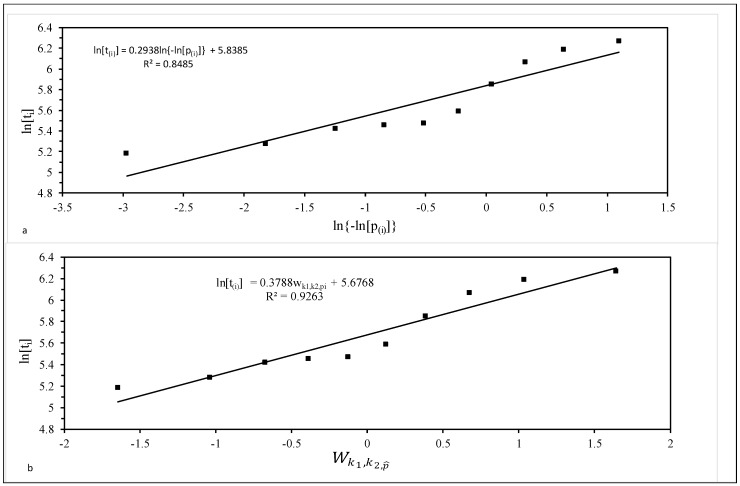
Probability plots for 10 batches of 1Cr-1Mo-0.25V tested at 823 K and 294 MPa: (**a**) the Weibull distribution; and (**b**) the log normal distribution.

As an illustration, Figure 4a is a $\ln(t_{i})/\;\ln\{-\ln[{\hat{p}}_{i}]\}$ plot for the *ten* 1Cr-1Mo-0.25V specimens tested at 823 K and 294 MPa. The best fit line obtained using the least squares technique is also shown. The slope of this best fit line is σ = 0.2938, with intercept μ = 5.8385. This implies β = 1/σ = 3.4 and λ = exp(−5.8385) = 0.0029. However, with a coefficient of determination (R^2^) of just 85%, the Weibull distribution is unlikely to be the best description of this sample of failure times.

This R^2^ value was computed over the range *p* = 0 to 2 and *q* = 0 to 1 (both in increments of 0.1), where

(11d) $$p=2(k_{1}+k_{2}{)}^{-1}\;\mathrm{and}\;q=(1/k_{1}-1/k_{2}){\left(1/k_{1}+1/k_{2}\right)}^{-0.5}$$

As such, this range covered all the distributions shown in Figure 3. It was found that R^2^ was maximised when *p* = *q* = 0, i.e., when *k*_1_ = *k*_2_ = ∞. It therefore appears that, within the generalised F distribution family, it is the log normal distribution that best describes the specimens tested at 823 K and 294 MPa. This is consistent with the findings by Evans [32].

Figure 4b plots ln(*t_i_*) against $w_{k1,k2,{\hat{p}}_{i}}$ when *k*_1_ = *k*_2_ = ∞, so that the variable on the horizontal axis is essentially a standard normal variate. The R^2^ value is 93% and so much higher than the Weibull case. The slope of this best fit line is *σ* = 0.3788 and can be interpreted as an estimate of the standard deviation in log times to failure. The intercept is *μ* = 5.6768 and can be interpreted as an estimate for the mean of the log times to failure at the stated test conditions. The survivor function associated with his normal distribution (using these parameter estimate) is shown in Figure 2. It tends to be lie above the parametric estimators at intermediate failure times, but below it as the higher failure times.

### 3.2. A Statistical Description of Continuous Failure Times at Varying Test Conditions

There are a number of approaches to extending the above concepts to the case of varying test conditions.

#### 3.2.1. Accelerated Failure Time Models (AFT)

In this type of model, μ in Equation (9b) is made a function of the test conditions

ln[*T*] = *Y* = μ + σ*W* = r(*x*) + σ*W* with

r(*x*) = r(*x*_1_ + *x*_2_ +…. + *x_m_*) (12a)

where *x*_1_ to *x_m_* are separate variables describing the test condition (for example, *x*_1_ may be stress, *x*_2_ temperature etc.) and r is an un-specified function (its form being best suggested by creep theory). *x* is a 1 by m matrix containing the *m* test variables that describe the test conditions for each of the N specimen placed on test. A commonly used specification for r(*x*) is

r(*x*) = b_1_*x*_1_ + b_2_*x*_2_ + … + b*_m_x_m_* (12b)

where b_1_ to b*_m_* are parameters that require estimation. As the name suggests, this approach has an accelerated life interpretation. In this formulation, the error term σ*W* is seen as a base or reference distribution that applies when *x*_1_ = *x*_2_ = … = *x_m_* = 0. This base distribution can be translated to a time scale by defining *T*_0_ = exp{σ*W*}. The probability that a test specimen will survive time *t*, S_0_(*t*), is then

S_0_(*t*) = Pr{*T*_0_ > *t*} = Pr{*W* > ln(*t*)/σ}

In this accelerated model, *T* is distributed as

*T*_0_exp(b_1_*x*_1_ + b_2_*x*_2_ + … + b*_m_x_m_*)

and so the test conditions act multiplicatively on survival times. Therefore, the probability that a test specimen with test conditions *x* will be survive time *t* is

S(*t*,*x*) = Pr{*T* > *t*|*x*} = Pr{*T*_0_e^r(**x**)^ > *t*} = Pr{*T*_0_ > *t*e^r(**x**)^} = S_0_(*t*e^b1*x*1+…+b*mxm*^) (12c)

Thus, the probability that a specimen with test conditions *x* will survive time *t* is the same as the probability that a base test specimen will be alive at time texp{r(*x*)}. This can be interpreted as time passing more rapidly by a factor exp{r(*x*)}—for example, twice as fast or half as fast. (A good analogy here is the use by humans of pet years to describe the age of their pets in relation to their life). Consider for example a multiplier of two for a specimen with test condition *x*. In terms of survival, this means that the probability that the specimen would be alive at any given time is the same as the probability that a base specimen would be alive at twice the length of time. In terms of risk, this model implies that an engineering component is exposed at any service life to double the risk of a base component that has been in service for twice as long.

The importance of Equation (12c) for this paper is that Evans [32] has shown, when using an AFT model, that whilst a generalised F distribution explained the shape of the failure time distribution at most test conditions for 1Cr-1Mo-0.25V steel, none of the distributions contained as special cases within the generalised F distribution adequately explained the shape of the actual failure time distributions at the remaining test conditions. This failure is explained by Equation (6c) as it shows that the survivor function should have the same form at all test conditions (namely that form identified for specimens tested at the base conditions)—unless time is stretched too much. However, in hazard based models, to be discussed below, the survivor function at a particular test conditions can differ markedly from that identified at the base test conditions and so offers extra flexibility over AFT models.

#### 3.2.2. Proportional Odds Models

Another approach assumes that the effect of the test conditions is to increase or decrease the odds of failure by a given duration by a proportionate amount:

(13a) $$\frac{1-S(t,x)}{S(t,x)}=\frac{1-S_{0}(t,x)}{S_{0}(t,x)}e^{b_{1}x_{1}+.....+b_{m}x_{m}}$$

where S_0_(*t*,*x*) is a baseline survivor function, taken from a suitable distribution, and exp{b_1_*x*_1_ + … + b*_m_x_m_*} is a multiplier reflecting the proportionate increase in the odds associated with test condition values *x*. Taking natural logs, gives

logit(1 − S(*t*,*x*)) = logit(1 − S_0_(*t*)) + b_1_*x*_1_ + … + b*_m_x_m_* (13b)

so the test conditions effects are linear in the logit scale. A somewhat more general version of the proportional odds model is known as the relational logit model. The idea is to allow the log-odds of failing in a given population to be a linear function of the log-odds in a reference or baseline population, so that

logit(1 − S(*t*)) = α + θlogit(1 − S_0_(*t*)) (13c)

The proportional odds model is the special case where θ = 1 (and where the constant α depends on the test conditions).

As an example, consider a proportional odds model with a log-logistic baseline. The corresponding survival function and the odds of failure are

(13d) $$S_{0}(t)=\frac{1}{{\left(\lambda t\right)}^{\beta }}\;;\;\frac{1-S_{0}(t)}{S_{0}(t)}={\left(\lambda t\right)}^{\beta }$$

Multiplying the odds by exp(b_1_*x*_1_ + … + b*_m_x_m_*) yields another log-logistic model. However, this is not true of other distributions: if the baseline survivor function is Weibull then this baseline multiplied by the odds of failing is not a Weibull survivor function.

#### 3.2.3. Proportional Hazard Models (PH)

The PH model of Cox [39] has a baseline hazard function h_0_(*t*) that shows how the hazard rate increases with time when this linear combination of test conditions equals unity

(14a) $$h(t|x)=h_{0}(t)\;r(x)$$

The log hazard function is then additive

(14b) $$\ln[h(t|x)]=\ln[h_{0}(t)]\;+\ln[r(x)]$$

Obviously, the cumulative hazards would follow the same relationship, as can be seen by integrating both sides of the previous equation. Exponentiating minus the integrated hazard, we find the survivor functions to be

S(*t*,*x*) = S_0_(t)^exp(b1*x*1+b2*x*2+…+b*mxm*)^ (14c)

so the survivor function for test conditions *x* is the baseline survivor raised to a power that is dependent upon the test condition. If a test specimen is exposed to twice the risk of a reference specimen at every point in time, then the probability that the specimen will be alive at any given time is the square of the probability that the reference or base specimen would be alive at the same time. In this PH model, a simple relationship in terms of hazards translates into a more complex relationship in terms of survival functions. Choosing a different parametric form for the baseline hazard, leads to a different model in the proportional hazards family. Apart from when the baseline hazard function corresponds to that of the Weibull hazard function, the hazard function at all other test conditions will be different in form from the baseline hazard function.

A possible limitation of the PH model is seen in Equation (14a), which implies that hazard functions associated with different test conditions are always constant multiples of one another—hence the name “proportional” hazards. One way to relax this proportionality assumption is to allow the test variables to interact with time or equivalently to allow b_1_, b_2_ etc. to be time dependent. Then, Equation (12b) becomes

b_1_(*tx*_1_) + b_2_(*tx*_2_) + … + b*_p_*(*tx_p_*) (15)

If, for example, the base line hazard function corresponds to the log normal distribution (so under test conditions r(*x*) = 1 the underlying failure time distribution is log normal), the underlying failure time distribution will not be log normal at any other test condition (i.e., when r(*x*) ≠ 1). This is a major advantage of building a failure time model around the hazard function rather than around the pdf or f(*t*).

### 3.3. Modelling Discrete Failure Times at Varying Test Conditions

Another major issue with hazard based models is to do with the identification of the baseline hazard function, h_o_(*t*). Without having many repeat tests carried out at a single test condition, it is difficult to accurately identify its functional form. One solution to this problem is to create a discrete failure time dataset from the original continuous one, i.e., split the continuous failure time data up into small but equally sized time spans. By doing so, it is possible to calculate a piecewise hazard function for each interval of time, which over all time intervals allows the shape of the base line hazard function to be identified. Springer and Willett [40] provide a good review of this approach. This is the approach taken in Section 4.

#### 3.3.1. Creating Discrete Data from Continuous Data

The first step required in building a discrete hazard function is to create the specimen-specimens dataset from the continuous failure time database. Here time is partitioned into *k* equal intervals I*_j_* = (a*_j_*_−1_ to a*_j_*), *j* = 1 to *k* and with *k* being as large as practically possible and a being a point in time. As an illustration of how this is done, consider batch VaA of the NIMS database, where *i* = 1, N = 43 specimens are tested, with each specimen receiving a different stress-temperature test combination. If *x*_1_ in Equation (12a) represent stress, then in batch VaA of NIMS this was varied from 412 MPa to 47 MPa and if *x*_2_ represents temperature this varied over the range 723 K to 948 K. The smallest recorded failure time was 338,760 s and the largest was 407,844,720 s. Many creep prediction models work with the log time to failure and so in natural log units these failure time limits corresponded to 12.73 and 19.83. The researcher then needs to decide upon how many discretised time intervals to work with. For example, the NIMS data could discretised into 15 equal (log) time intervals, respectively giving (log) time intervals of width 0.5. In this example, k = 15 and a*_j_*_−1_ − a*_j_* = 0.5 with a_0_ = 12.5 and a*_k_* = 20). The first interval this NIMS dataset is therefore 12.5–13.0 and corresponds to *j* = 1, the second is 13.0–13.5 and corresponding to *j* = 2 all the way up to the interval 19.5–20 and corresponding to *j* = 15. The specimens-specimen data are then generated by creating a binary variable, v, for each time interval. Thus, the binary variable equals 0 in time interval I*_t_* if the specimen does not fail in that interval and 1 if it does. This binary variable is created for each specimen in the test matrix.

Table 1 illustrates the start of the creation of this specimens-specimen format by considering just the firsts two NIMS specimen in batch VaA using a*_j_*_−1_ − a*_j_* = 0.5. The first specimen was tested at *x*_1_ = 412 MPa and *x*_2_ = 723 K. It failed at 16.36 log seconds. The second was tested at *x*_1_ = 373 MPa and *x*_2_ = 723 K and it failed at 17.84 log seconds. Continuing the process shown in Table 2, creates M values for v, where M = kN = 358 for this NIMS batch.

#### 3.3.2. Re-Specification of the Continuous Hazard Based Models

Equation (14b) can be re-specified as

(16a) $$\ln[h(ij|x)]={\ln[h}_{0}(j)]\;+\ln[\;r(x_{i})]=b_{0,j}D_{j}+b_{1}x_{1i}+\ldots +b_{m}x_{mi}$$

where ${\ln[h}_{0}(j)]\;=b_{0,j}{\;\mathrm{and}\;D}_{j}={\;1\;\mathrm{if}\;v}_{ij}={1\;\mathrm{in}\;\mathrm{interval}\;a}_{j-1}-a_{j}{\;\mathrm{and}\;D}_{j}\;=\;0\;\mathrm{otherwise}\;(j=1,..,k)$.

In Equation (14b), *x* is a Nk by 2 matrix where each column contains the *i* different stress and temperature combinations that each specimen was tested at and *x_i_* is the *i*th first row of the matrix *x*. However, when the data are discretised in this way, h(*ij*|*x*) in Equation (14a) is not directly observable. Instead, there is the binary variable v*_ij_* that equals zero when the specimen is un-failed in time interval a*_j_*_−1_ − a*_j_* or 1 if it fails in that time interval. Therefore, what is required is a non-linear function that maps between v*_ij_* = 0 and v*_ij_* = 1 to give the hazard rate between 0 and 1 for each specimen in each time interval. Two commonly used functions that achieve this are the logistic

(16b) $$v_{ij}=[h(ij|x)]=\frac{e^{b_{0,j}D_{j}+b_{1}x_{1i}+\ldots +b_{m}x_{mi}}}{1+e^{b_{0,j}D_{j}+b_{1}x_{1i}+\ldots +b_{m}x_{mi}}}\;$$

and the log-log

(16c) $$v_{ij}=[h(ij|x)]=1-\exp\left\{-e^{b_{0,j}D_{j}+b_{1}x_{1i}+..+b_{m}x_{mi}}\right\}$$

functions. These are in turn special cases of a more general S shaped curve given by

(16d) $$v_{ij}=[h(ij|x)]=1-{\left\{1+(1/\alpha {)\exp(b}_{0,j}D_{j}+b_{1}x_{1i}+\ldots +b_{2}x_{mi})\right\}}^{-\alpha }$$

so Equation (16d) collapses to Equation (16b) when α = 1 and tends to Equation (16c) as α tends to ∞.

Plotting estimated values of b_0,*j*_ against time allows the shape of the baseline hazard function to be observed, which in turn could lead to a simplification of Equation (16d). For example, if such a plot reveals a straight line, the k b_o,*j*_D*_j_* terms can be replaced by a + b_0_*j*

(16e) $$v_{ij}=[h(ij|x)]=1-{\left\{1+(1/\alpha )\exp(a+b_{0}j+b_{1}x_{1i}+\ldots +b_{2}x_{mi})\right\}}^{-\alpha }$$

Non-proportionality can then be accommodated by allowing the *x* variables to interact with time t in the following way

(16f) $$v_{ij}=[h(ij|x)]=1-{\left\{1+(1/\alpha )\exp(a+b_{0}j+b_{1}jx_{1i}+....+b_{2}jx_{mi})\right\}}^{-\alpha }$$

#### 3.3.3. Estimation

These discrete hazard models are essentially binary response regression models and so the unknown parameters can be estimated by maximising the log likelihood function given by

(17) $$\ln\{L\}=\sum_{i=1}^{N}\sum_{j=1}^{k}\left[v_{ij}\ln\left(\frac{h(ij|x)}{1-h(ij|x)}\right)\right]+\sum_{i=1}^{N}\sum_{j=1}^{k}\left(1-v_{ij})(\ln(1-h(ij|x\right)$$

where the binary response variable v*_ij_* and h(*t*|*x*) is given by Equation (16d), Equation (16e) or Equation (16f). Hence, Equation (17) will be maximised yielding some given set of values for the unknown parameters on the right hand side of Equation (16d), Equation (16e) or Equation (16f). This function can be maximised for various values of α, with the chosen value for α corresponding to the largest of these maximised log likelihoods. Direct maximisation can be carried out using standard non-linear optimisation algorithms [41], or alternatively Equation (17) can be maximised indirectly by using the iteratively reweighted least squares algorithm of McCullagh and Nelder [42].

#### 3.3.4. Assessing Model Adequacy

There are a number of ways to assess the adequacy of these discrete hazard based models. The discrete hazard models given by either Equation (16d), Equation (16e) or Equation (16f) produce a predicted hazard rate or probability of failure within the time intervals a*_t_*_−1_ to a*_t_*, rather than a specific time at which failure occurs. This makes it a little more complicated to assess whether the model is capable of accurately predicting the observed failure times. To obtain predicted times to failure (more specifically the time interval in which failure is predicted to occur), a cut-off point c is needed, such that if the predicted hazard rate exceeds c in time interval a*_t_*_−1_ to a*_t_* then failure is predicted to have occurred in that time zone. Otherwise, the prediction is that the specimen will not fail in that time interval. The usual value for c is 0.5, and once chosen a simple classification table, such as that in Table 2, can be constructed.

In Table 2, there are M_2_ time intervals where a specimen failed. The model correctly predicted correctly m_4_ of these, but incorrectly predicted m_3_ of these intervals, that is m_3_ specimens failed in times zones different from those the model predicted them to fail in (where M_2_ = m_3_ + m_4_). Similarly, there are M_1_ time zones where v*_ij_* actually equalled zero (M_1_ time zones not containing failed specimens) and of these, the model predicted correctly m_1_ of these time zones, but incorrectly predicted m_2_ of these zones (i.e., there were m_2_ times zones where a specimen survived, but the model predicted failures to occur). M can be found from summing either M_1_ and M_2_ or M_3_ and M_4_.

The models success rate in predicting the time zones where specimens will not fail is given by m_1_/M_1_. This can be taken to be the probability of the model correctly detecting a false signal and is called the models specificity. The models success rate in predicting the time zones where specimens will fail is given by m_4_/M_2_. This can be taken to be the probability of the model correctly detecting a true signal and is called the models sensitivity. The models over-all rate of correct classification is then given by (m_1_ + m_4_)/M.

Given the way in which a specimens-specimen dataset is constructed, there are many more values of v*_ij_* = 0 compared to v*_ij_* = 1, it is common to observe sensitivity values well below specificity values. Thus a well specified model will have high values for both sensitivity and specificity. However, the sensitivity and specificity depend in part on the chosen value for the cut-off point c and c = 0.5 may not be the optimal value as far as failure time prediction is concerned. One solution to this is to construct a classification table for a range of cut-off points to see how the discrete hazard model works as a classifier of when failure will occur. However, a neater way of doing this is given by the area under the receiver operating characteristic (ROC) curve. The ROC emerges on a graph that plots the sensitivity against (1-specificity) associated with all possible values for c (c = 0 to 1). The resulting area under the ROC curve lies between zero and unity and measures the ability of the hazard model to discriminate between time zones where a specimen will fail and zones where it will not. Hosmer and Lemenshow [43] suggest the following benchmarks for this area: An ROC of 0.5 provides no discrimination implying the model performance no better than tossing a coin to decide if a specimen fails in a given time interval. An ROC between 0.7 and 0.8 gives acceptable discrimination, whilst a ROC between 0.8 and 0.9 gives excellent discrimination. Finally, a ROC above 0.9 gives outstanding discrimination.

One way to determine an optimal value for c is to choose that value that yields the best failure time predictions. For example, if c inserted for v in say Equation (16e), then the variable t on the right hand side of the equation becomes the predicted time zone at which a specimen fails, $\hat{t}$

(18a) $$c=[h(ij|x)]=1-{\left\{1+(1/\alpha )\exp(a+b_{0}\hat{j}+b_{1}x_{1i}+\ldots +b_{2}x_{mi})\right\}}^{-\alpha }$$

This can be solved for $\hat{j}$

(18b) $$\hat{j}=\{\ln[{\left(1-c\right)}^{\left(-1/\alpha \right)}-1]+\ln(\alpha )-(a+b_{1}x_{1i}+\ldots +b_{2}x_{mi})\}/b_{0}$$

Thus, $\hat{j}$ is a prediction of the time interval where a specimen fails. For example, if $\hat{j}$ = 2.5, failure is predicted to occur in the time interval I_2_ = a_2_ − a_1_, and the actual predicted time is then equal to (a_2_ + a_1_)/2 (or another example, actual failure time = 0.25a_2_ + 0.75a_1_ if $\hat{j}$ = 2.75). Notice the *j* = 2 time interval is, from Table 1, the 12.5–13 logged seconds and its mid point is therefore 12.75 logged seconds (or 96 h).

A plot can then be made of actual failure time against this predicted time. If a best fit line is put through the data on such a plot, the optimal value for c can be taken to be the one that produces a best fit line closest to the 45° line on such a plot. As an alternative, c can be chosen to minimise the mean squared error defined as

(18c) $$\mathrm{MSE}=\frac{{\left[j-\hat{j}\right]}^{2}}{N}$$

Using c = 0.5 in Equation (18b) can also be interpreted as yielding a median predicted time to failure, whilst using c = 0.05 produces a time interval prediction such that there is only a 5% chance of failure occurring in that or an early time interval. Likewise using c = 0.95 produces a time interval prediction such that there is a 95% chance of failure occurring in that or an early time interval. These then come together to define a 90% confidence interval for the time interval where failure will occur.

## 4. Application of Discrete Hazard Function to Batch VaA of 1Cr-1Mo-0.25V

### 4.1. Incorporating Wilshire Variables into a Discrete Hazard Model

The intention of this paper is to keep within the discrete hazard model as many features of the Wilshire methodology as possible—purely to illustrate the assimilation of that creep model with this statistical model for the random component of creep and not because one implies the other. In its simplest form, the Wilshire Equation is given by Equation (5b). However, the hazard function describes a failure rate or conditional probability of failure and not the failure time itself. There are a number of ways to incorporate this Wilshire equation into a discrete hazard based model and the following describes some of the possibilities. The starting point is to map Equation (5b) onto Equation (16d–f) by replacing ln[*t*] = y with the log hazard rate ln[h(*t*|*x*)]. This gives, based on Equation (16d),

(19a) $$\ln[h(ij|x)]={\ln[h}_{0}(j)]\;+\ln[\;r(x_{i})]=b_{0,j}D_{j}+b_{1}x_{1i}+b_{2}x_{2i}\;$$

where *x*_1_ = τ* and *x*_2_ = 1000/RT.

However, h(ij|*x*) is not observable. Instead there is the binary variable v*_ij_* that equals zero when the specimen is un-failed in time interval a*_j_*_−1_ − a*_j_* or 1 if it fails in that time interval. Therefore, what is required is a non-linear function that maps between v*_ij_* = 0 and v*_ij_* = 1 to give the hazard rate between 0 and 1 for each specimen in each time interval. Section 3.3.2 outlined a general form of such a function allowing Equation (19a) to be written as

(19b) $$v_{ij}=[h(ij|x)]=1-{\left\{1+(1/\alpha {)\exp(b}_{0,j}D_{j}+b_{1}x_{1i}+b_{2}x_{2i})\right\}}^{-\alpha }$$

If the b_0,*j*_ parameters trace out a linear time trend, then Equation (19b) can be written as

(19c) $$v_{ij}=[h(ij|x)]=1-{\left\{1+(1/\alpha )\exp(a+b_{o}j+b_{1}x_{1i}+b_{2}x_{2i})\right\}}^{-\alpha }$$

As will be seen below, this plausibility of such a simplification can be assessed by plotting out the estimated b_0*j*_ values. Non-proportionality can then be accommodated by allowing *τ* and 1000/RT to interact with time in the following way

(19d) $$v_{ij}=[h(ij|x)]=1-{\left\{1+(1/\alpha )\exp(a+b_{0}j+b_{1}jx_{1i}+b_{2}jx_{2i})\right\}}^{-\alpha }$$

In addition, it is known (see Wilshire [21] and Evans [32]) that for this material the relationship between t and τ* changes at some critical value for τ* (i.e., b_1_ changes value at this point) and that the activation energy changes at around 823 K (i.e., b_2_ changes value at this point). Thus, Equation (19b) can be written as

(19e) $$v_{ij}=[h(ij|x)]=1-\{1+(1/\alpha {)\exp(b}_{0,j}D_{j}+b_{1}x_{1i}+b_{2}x_{2i}+b_{3}x_{1i}B_{1i}+b_{4}x_{2i}B_{2i}{\}}^{-\alpha }$$

where B_1_ = 0 when τ* ≤ *τ*_crit_ and unity otherwise. Similarly, B_2_ = 0 when *T* ≤ 823 and unity otherwise. The reason for doing this is that it allows stress and temperature to have a different effect on the hazard rate either side if 823 K and *τ*_crit_. The explanation provided by Wilshire is that as *τ*_crit_ is close in value to the yield stress, dislocation movement is confined to grain boundaries below the yield stress so that the activation energy falls below that for self diffusion through the crystal—so causing the value for b_2_ to change. Thus, when τ* ≤ *τ*_crit_, B_2_ = 0, and so the effect of temperature on the hazard rate is determined by the value for b_2_. However, when τ* > *τ*_crit_, B_2_ = 1, and so the effect of temperature on the hazard rate is determined by the value for b_2_ + b_4._ The role of stress is also different either side of *τ*_crit_ (changing from b_2_ to b_2_ + b_3_).

Often, the values for b_0,*j*_ reveal a linear trend or some well defined non-linear trend such as a polynomial, exponential or power law trend. For example, if a linear trend is revealed by a plot of the b_0,*t*_ values against t, then Equation (19e) takes the form

(19f) $$v_{ij}=[h(ij|x)]=1-\{1+(1/\alpha )\exp(a+b_{0}j+b_{1}x_{1i}+b_{2}x_{2i}+b_{3}x_{1i}B_{1i}+b_{4}x_{2i}B_{2i}{\}}^{-\alpha }$$

In Equation (19e,f), the effect of changing test conditions is to shift in a parallel fashion the log baseline hazard function, but it is also possible to allow the slope of the baseline hazard function to depend on *x*_1_ and/or *x*_2_. For example, if b_0_ depends on *x*_1_ then Equation (19f) becomes

(19g) $$v_{ij}=[h(ij|x)]=1-\{1+(1/\alpha )\exp(a+b_{0}j+b_{1}x_{1i}+b_{2}x_{2i}+b_{3}x_{1i}B_{i1}+b_{4}x_{2i}B_{2i}+b_{5}x_{1i}j{\}}^{-\alpha }$$

### 4.2. Results

#### 4.2.1. Model Given by Equation (19e)

A specimens-specimen dataset was created for batch VaA using *k* = 15 and I*_j_ =* a*_j_*_−1_ − a*_j_* = 0.5 with a_0_ = 12.5 and a*_k_* = 20. This dataset consisted of M = 358 observations on v. Using these data, the parameters of Equation (19e) were estimated for a range of values for α and *τ*_crit_. The values for α and *τ*_crit_ that produced the highest ROC were α = 1 and *τ*_crit_ = 0.1. This α value suggests the logistic discrete hazard model is preferable to the log-log discrete hazard model, whilst the *τ*_crit_ value is a little higher than that identified by Evans [32] and Wilshire [21] but is broadly similar in value. Table 3 shows the results of applying the McCullagh and Nelder algorithm to this data. The values for *x*_1_ and *x*_2_ were normalised to be zero at 823 K and 294 MPa so that the resulting estimated value for b_0,*t*_ give the baseline log hazard rates that corresponds to this test condition.

The student *t*-values associated with *x*_1_ and *x*_2_ in Table 3 reveals that both τ* and the reciprocal of temperature are statistically significant variables so yielding support for the Wilshire methodology, whilst the last two rows show a discontinuity in the Wilshire model at τ* = 0.1 and at a temperature of 823 K. These estimates are not comparable in value to those in Equation (5b) because the latter show the impact of τ* and *T* on failure times directly, rather than the log hazard rate as is so in the former case. Reading across row one of Table 3, the estimated log hazard rate for time interval *j* = 1 (12.5–13.0 log seconds) when temperature is 823 K and stress is 294 MPa is −4.7526, implying a hazard rate of exp(−4.7526) = 0.0086. This hazard rate corresponds to the log time interval of 12.5 to 13.0, which in turn corresponds to a time interval in hours of 75 to 123. Associating this hazard rate with the mid point of this time interval gives a hazard rate of 0.0086 at time 100 h. Proceeding in the same way for this next two rows of Table 3 gives a cumulative hazard rate of 0.189 at 163 h and 0.795 at 268 h. Recall that Figure 2 show the non-parametric estimate of the cumulative hazard rate associated with the test conditions of this estimated baseline hazard rate. Reading of this graph at 270 h shows the non-parametric estimate of the cumulative hazard rate is 0.85, showing this model is consistent with this non-parametric estimator.

**Figure 5:**
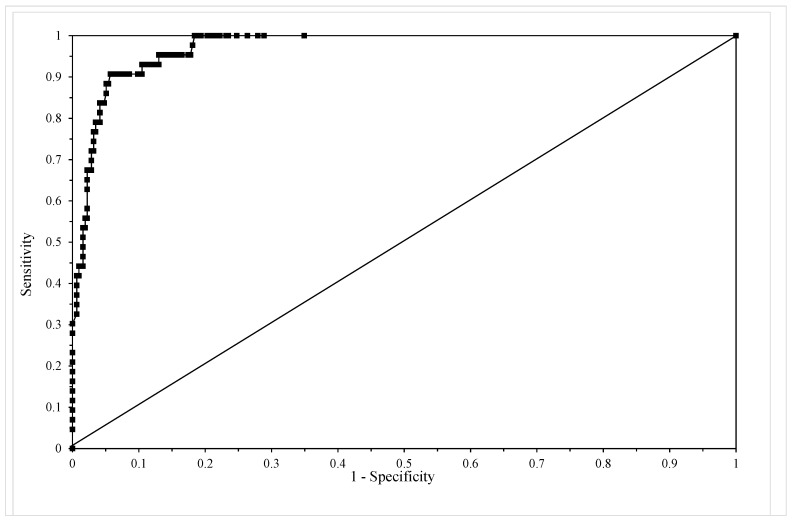
The receiver operating characteristic (ROC) from model given by Equation (13e).

Table 4 shows the classification table for this model using c = 0.5 revealing that sensitivity equals 68% and specificity equals 98% with an overall correct failure time zone prediction rate of 94%. Figure 5 shows the ROC for this model and the area under this curve is 0.972, which according to Hosmer and Lemenshow, makes this model outstanding in its ability to discriminate between failure and non-failure in the 15 time zones. 

**Figure 6:**
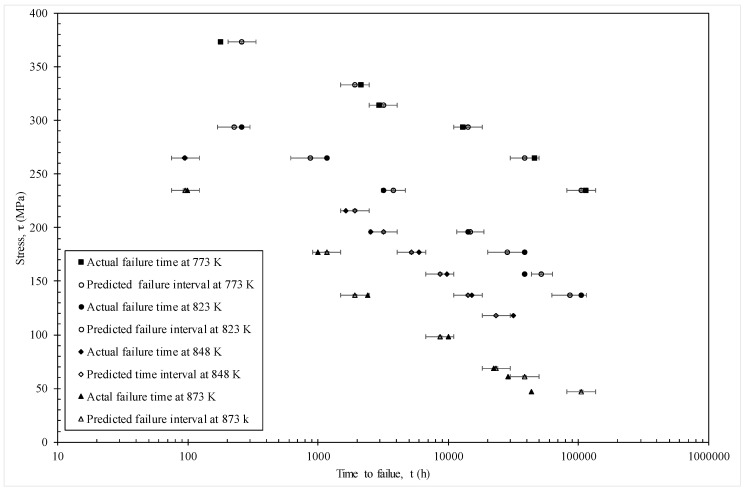
Actual failure times and predicted times interval obtained from the model given by Equation (19e).

The MSE is minimised at c = 0.53 with a value of 0.0526. Figure 6 then plots the actual times to failure (hours) for specimens in batch VaA, together with the failure time zones predicted by the model using c = 0.53. The error bars shown around the predictions reflect the fact that this model predicts the time interval in which failure occurs and the width of this time interval is 0.5 log hours. The models predictions are taken to be the mid points of these error bars. With only a few exceptions (for example, at 773 K with 373 MPa and 823 K with 157 MPa), the model predicts the time interval at which failure actually occurs correctly. The worst prediction comes at 873 K and 47 MPa—but this point was also poorly predicted in the original Wilshire [21] paper as well.

**Figure 7:**
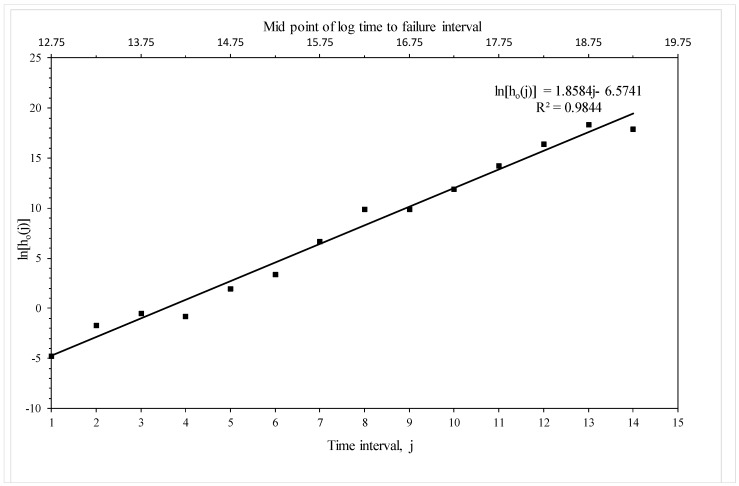
Estimated baseline (i.e., corresponding to 823 K and 294 MPa) piece-wise log hazard function based on Equation (19e).

Figure 7 plots the piece-wise log hazard rates associated with each time zone (the b_0*t*_ in Table 3) against the numbered time zone and this plot reveals a well-defined linear trend. The trend is very strong with an R^2^ value of 98.4% with no obvious deviation from linearity. This suggests that it should be possible to create a more parsimonious version of Equation (19e), without affecting the predictive ability of the simpler model, by replacing the fifteen b_0*t*_ parameters with a linear trend containing just two parameters—a and b_0_. Figure 7 implies that a = 6.57 and b_0_ = 1.86. This parsimonious version is estimated in the following subsection.

#### 4.2.2. Model Given by Equation (19f)

When Equation (19g) was estimated, the t value associated with parameter b_5_ suggested that b_5_ was insignificantly different from zero at the 1% significance level so that the data were not supportive of the slope of the log base hard function estimated in the previous sub section changing at some critical value for the normalised stress. Table 5 shows the estimated parameters of Equation (19f) obtained using the McCullagh and Nelder algorithm (again the values for α and *τ*_crit_ that produced the highest ROC were α = 1 and *τ*_crit_ = 0.1).

The estimated values for b_1_ to b_4_ in Table 5 are consistent with those shown in Table 3 and, again, the student *t*-values associated with these parameters reveal they are significantly different from zero at either the 1% or 5% significance level. Further, the values for a and b_0_ are not that dissimilar from the values sown in Figure 6. These parameter estimates can be used to calculate the hazard rate at the base or reference test conditions of temperature = 823 K and stress = 294 MPa. For example, in time zone *j* = 1 (corresponding to the log time interval of 12.5 to 13 or 75 to 123 h) the hazard rate is predicted to be exp(−5.8351 + 2.0736 × 1) = 0.0232.

**Figure 8:**
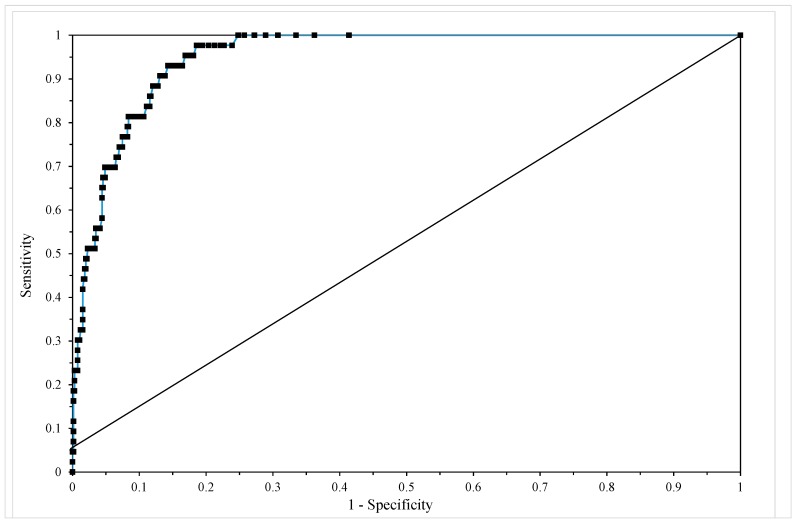
The receiver operating characteristic (ROC) from model given by Equation (19f).

Table 6 shows the classification table for this model when c = 0.5 revealing that sensitivity equals 69.8% and (1-specificity) equals 96.8% with an overall correct failure time zone prediction rate of 95%. Figure 8 shows the ROC for this model and the area under this curve is 0.951, which according to Hosmer and Lemenshow, makes this model outstanding in its ability to discriminate between failure and non-failure in the 15 time zones. Further, this value is only slightly below that from the previous model, showing that the use of a simple linear time trend instead of a piece-wise hazard function has not resulted in a significant reduction in predictive ability.

**Figure 9:**
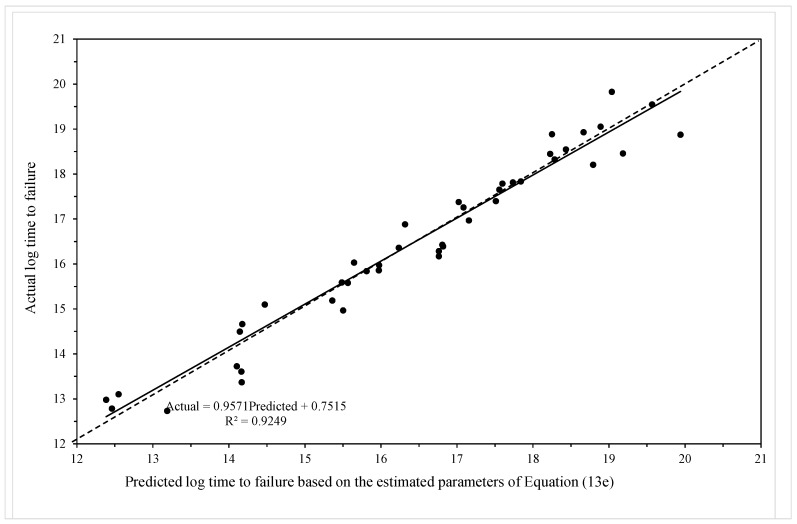
Actual log failure times plotted against predicted log failure times obtained from the model given by Equation (19f).

The MSE is minimised at c = 0.53 and Figure 9 then plots the actual times to failure for specimens in batch VaA against the failure time predicted by the model using c = 0.53. As a continuous base hazard function is now used instead of the piece-wise hazard function, an actual failure time, rather than an interval time, prediction can be made. Figure 9 plots the actual failure times against the predicted failure times (n natural logs). The best fit line on this plot is very close to the ideal outcome associated with the 45 degree line—corresponding to a situation where the model predicts each failure time perfectly. Hence, the bias in prediction remains small in this more parsimonious model.

**Figure 10:**
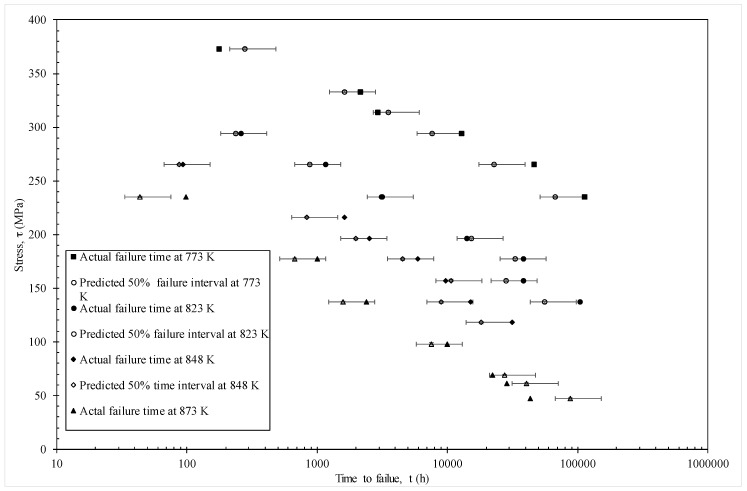
Actual failure times and predicted failure times obtained from the model given by Equation (19f).

Figure 10 shows a different representation of these predictions—where stress is shown on the vertical axis and times to failure on the horizontal. This time the error bars show a 50% prediction band based on using c = 0.25 and c = 0.75 in Equation (18b). Again, and with only a few exceptions, the actual failure times fall within the models 50% prediction bands.

**Figure 11:**
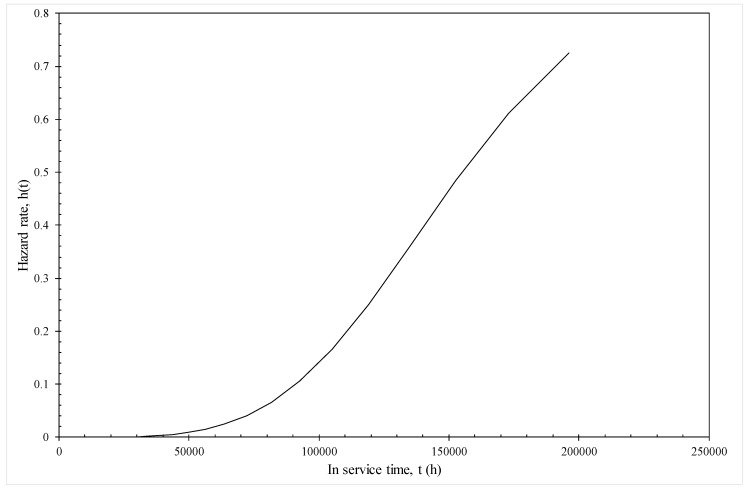
Predicted hazard rates at various times in operation at 823 K and 130 MPa from the model given by Equation (19f).

Figure 11 illustrates how this model can be used to predict the hazard rates associated with in service life under various conditions—in this illustration operating at 130 MPa and 823 K. It can be seen that the hazard rate remains very close to zero up to 50,000 h or about 8 years of in service use. The risk of failure then starts to rise quite dramatically. For example, if this material had been in operation for around 15 years, the chances of it failing in the next year is around 35%, but, if this material had been in operation for around 25 years, the chances of it failing in the next three years rises dramatically to around 80%.

## 5. Conclusions

This paper has provided a summary review of some statistical failure time models with the aim of aiding the assimilation of such models with existing predictive models for creep life. This will enable an enrichment of prediction to be achieved with a move away from predicting failure times on the average towards predicting the safe life associated with a minimum chance of failure This was followed by an illustration of one possible assimilation, namely—the deterministic Wilshire equation and the statistical discrete hazard model. This statistical model was chosen because it provided the capability of estimating failure probabilities in future time intervals for materials that have already been in service for various lengths of time.

Estimation of this model revealed that at a fixed test condition, the log of the probability of failure in the next time interval (given survival up to then) is a linear function of time. This log base line hazard function then shifted in a parallel fashion with the well-known Wilshire variables of τ* and the reciprocal of temperature. Like in the original Wilshire methodology, this shifting nature of the base hazard function was different above and below 823 K and τ* = 0.1. The model was shown to produce outstanding discrimination with respect to which time interval a specimen would fail in. Finally, and as an illustration of the output this model was capable of producing, it was found that if this material had been in operation for around 15 years at 823 K and 130 MPa, the chances of it failing in the next year is around 35%. However, if this material had been in operation for around 25 years under this condition, the chances of it failing in the next year rose dramatically to around 80%.

## Figures and Tables

**Table 1:** Illustration of the creation of a specimens-specimen dataset.

Time Interval I*_j_* = a*_j_*_−1_ − a*_j_* (Log Seconds)	Specimen Number, *i*	Stress, *x*_1_ (MPa)	Temperature, *x*_2_ (K)	v*_ij_*
12.5–13.0	1	412	723	0
13.0–13.5	1	412	723	0
13.5–14.0	1	412	723	0
14.0–14.5	1	412	723	0
14.5–15.0	1	412	723	0
15.0–15.5	1	412	723	0
15.5–16.0	1	412	723	0
16.0–16.5	1	412	723	1
12.5–13.0	2	373	723	0
13.0–13.5	2	373	723	0
13.5–14.0	2	373	723	0
14.0–14.5	2	373	723	0
14.5–15.0	2	373	723	0
15.0– 15.5	2	373	723	0
15.5–16.0	2	373	723	0
16.0–16.5	2	373	723	0
16.5–17.0	2	373	723	0
17.0–17.5	2	373	723	0
17.5–18.0	2	373	723	1

**Table 2:** Classification table for a discrete hazard model.

Predicted v*_ij_*	Observed v*_ij_*		Total
	Survived: v*_ij_* = 0	Failed: v*_ij_* = 1	
Survived: v*_ij_* = 0	m_1_	m_3_	M_3_
Failed: v*_ij_* = 1	m_2_	m_4_	M_4_
Total	M_1_	M_2_	M

**Table 3:** Estimation of the parameters in Equation (19e).

Parameter	Variable	Estimate	Student *t*-value
b_0,1_	ln[h_o_(*j* = 1)] for 12.5–13.0	−4.7526	−2.81 **
b_0,2_	ln[h_o_(*j* = 2)] for 13.0–13.5	−1.7111	−1.43
b_0,3_	ln[h_o_(*j* = 3)] for 13.5–14.0	−0.5019	−0.48
b_0,4_	ln[h_o_(*j* = 4)] for 14.0–14.5	−0.7838	−0.53
b_0,5_	ln[h_o_(*j* = 5)] for 14.5–15.0	1.9713	1.26
b_0,6_	ln[h_o_(*j* = 6)] for 15.0–15.5	3.4266	2.16 **
b_0,7_	ln[h_o_(*j* = 7)] for 15.5–16.0	6.7247	4.24 ***
b_0,8_	ln[h_o_(*j* = 8)] for 16.0–16.5	9.8840	4.81 ***
b_0,9_	ln[h_o_(*j* = 9)] for 16.5–17.0	9.9378	4.31 ***
b_0,10_	ln[h_o_(*j* = 10)] for 17.0–17.5	11.9647	4.85 ***
b_0,11_	ln[h_o_(*j* = 11)] for 17.5–18.0	14.2367	5.11 ***
b_0,12_	ln[h_o_(*j* = 12)] for 18.0–18.5	16.4289	5.27 ***
b_0,13_	ln[h_o_(*j* = 13)] for 18.5–19.0	18.3696	5.39 ***
b_0,14_	ln[h_o_(*j* = 14)] for 19.0–19.5	17.9018	5.04 ***
b_0,15_	ln[h_o_(*j* = 15)] for 19.5–20.0	19.2411	0.38
b_1_	*x*_1_	−28.554	−5.13 ***
b_2_	*x*_2_	−1363.8818	−5.62 ***
b_3_	*x*_3_	5.4672	2.37 **
b_4_	*x*_4_	642.0808	−4.43 ***

Parameters estimates using the iteratively reweighted least squares technique of McCullagh and Nelder [42]. Student *t*-values test the null hypothesis that the true parameter values equal zero. ** identifies statistically significant variables at the 5% significance level, and *** identifies statistically significant variables at the 1% significance level. These levels of significance are based on the student *t*-statistic that has a student t distribution.

**Table 4:** Classification table for a discrete hazard model of Equation (13e) with c = 0.5.

Predicted v*_ij_*	Observed v*_ij_*		Total
	Survived: v*_ij_* = 0	Failed: v*_ij_* = 1	
Survived: v*_ij_* = 0	306	14	320
Failed: v*_ij_* = 1	9	29	38
Total	315	43	358

**Table 5:** Estimation of the parameters in Equation (19f).

Parameter	Variable	Estimate	Student *t*-value
a	Constant	−5.8351	−4.91 ***
b_0_	Time trend, *j*	2.0736	6.02 ***
b_1_	*x*_1_	−30.8703	−5.85 ***
b_2_	*x*_2_	−1451.7496	−5.93 ***
b_3_	*x*_3_	5.1666	3.84 ***
b_4_	*x*_4_	596.9284	4.48 ***

Parameters estimates using the iteratively reweighted least squares technique of McCullagh and Nelder [42]. Student *t*-values test the null hypothesis that the true parameter values equal zero. *** identifies statistically significant variables at the 1% significance level. These levels of significance are based on the student *t*-statistic that has a student t distribution.

**Table 6:** Classification table for a discrete hazard model of Equation (19f) with c = 0.5.

Predicted v*_ij_*	Observed v*_ij_*		Total
	Survived: v*_ij_* = 0	Failed: v*_ij_* = 1	
Survived: v*_ij_* = 0	305	13	318
Failed: v*_ij_* = 1	10	30	40
Total	315	43	358

## Appendix A. The Generalised F Family of Failure Time Distributions

Using the change of variables technique, and substituting *Z* = (*Y* − μ)/b into the pdf of Equation (9c) in Section 3.1.2 yields

(A1) $$f(y)=f(z)\frac{\partial z}{\partial y}=\frac{1}{b}\frac{{\left(k_{1}/k_{2}\right)}^{k_{1}}}{\left\{\Gamma {(k}_{1})\Gamma {(k}_{2})\right\}/{\Gamma (k}_{1}+k_{2})}\frac{\exp\;\left(\frac{k_{1}}{b}[y-\mu ]\right)\;}{{\left(1+\left\{k_{1}/k_{2}\right\}e^{\frac{k_{1}}{b}[y-\mu ]}\right)}^{\left(k_{1}+k_{2}\right)}}$$

Using β = 1/b, λ = exp(−μ) and *T* = exp(*Y*), it follows that

(A2) $$\exp\left(\frac{k_{1}}{b}[y-\mu ]\right)=\exp{\left(y\right)}^{k_{1}/b}\exp{\left(-\mu \right)}^{k_{1}/b}=t^{{}^{k_{1}/b}}\lambda^{k_{1}/b}={\left(\lambda t\right)}^{k_{1}/b}={\left(\lambda t\right)}^{k_{1}\beta }$$

Substituting Equation (A2) into Equation (A1) and using the change of variable technique then gives the pdf for *T*

(A3) $$\begin{array}{cc} f(t) & =f(y)\frac{\partial y}{\delta t}=\frac{t^{-1}}{b}\frac{{\left(k_{1}/k_{2}\right)}^{k_{1}}}{\left\{\Gamma (k_{1})\Gamma (k_{2})\right\}/\Gamma (k_{1}+k_{2})}\frac{{\left(\lambda t\right)}^{k_{1}\beta }}{{\left(1+\{k_{1}/k_{2}\}{\left(\lambda t\right)}^{k_{1}\beta }\right)}^{\left(k_{1}+k_{2}\right)}}\\ & =\frac{{\left(k_{1}/k_{2}\right)}^{k_{1}}\lambda \;\beta }{\left\{\Gamma (k_{1})\Gamma (k_{2})\right\}/\Gamma (k_{1}+k_{2})}\frac{{\left(\lambda t\right)}^{\beta \;k_{1}-1}}{{\left(1+\{k_{1}/k_{2}\}{\left(\lambda t\right)}^{k_{1}\beta }\right)}^{\left(k_{1}+k_{2}\right)}} \end{array}$$

This equation is not valid when *k*_1_ = *k*_2_ = ∞, which corresponds to the log normal distribution. Abramowitz and Stegun [44] show that the gamma function can be approximated by

(A4) $$\ln[\Gamma (k)]=(k-0.5)\ln(k)-k+0.5\ln(2\pi )+\frac{1}{12k}-\frac{1}{360k^{3}}+\frac{1}{1260k^{5}}-\frac{1}{1680k^{7}}+\ldots$$

and based on this approximation, it follows that Г (*k* + 1) = *k*Г(*k*) and Г(1) = 1. As mentioned in the main text, *Z* is a standardised variable, and its mean (or expected value E) and its variance are given by

E[*Z*] = Ψ(*k*_1_) − Ψ(*k*_2_) + ln(*k*_2_) − ln(*k*_1_) (A5)

Var[*Z*] = Ψ^/^(*k*_1_) + Ψ^/^(*k*_2_) (A6)

where Var(*Z*) reads the variance of *Z* and the di (Ψ) and tri (Ψ^/^) gamma functions are the first and second derivatives respectively of the log gamma function with respect to *k* and so

(A7) $$\Psi (k)=\ln(k)-\frac{1}{2k}-\frac{1}{12k^{2}}+\frac{1}{120k^{4}}-\frac{1}{252k^{6}}+\ldots$$

(A8) $$\Psi^{/}(k)=\frac{1}{k}+\frac{1}{2k^{2}}+\frac{1}{6k^{3}}-\frac{1}{30k^{3}}+\frac{1}{42k^{7}}+\ldots$$

The distribution of *Z* is degenerate in that as either *k*_1_ or *k*_2_ tends to infinity, Ψ^/^(*k*) tends to zero and so the variance of *Z* tends also to zero, i.e., the pdf for *Z* collapses to a single point. However, from the rules behind computing expected values, it follows that Var(*W*) = δ^2^Var(*Z*).

For example, as *k*_2_ → ∞, δ^2^ → *k*_1_, Ψ^/^(*k*_2_) → 0 and Ψ^/^(*k*_1_) → 1/*k*_1_ and so Var[W] → 1. Thus, W has a non-degenerate distribution.

It follows from Equations (A7) and (A8) and the rules of expected values that:

E[*Y*] = μ + bE[*Z*] (A9)

and

Var(*Y*) = b^2^Var(*Z*) (A10)

where Var(*Y*) is the variance of *Y*. Meeker and Escobar [45] have shown that, when *k*_2_ > 1/β the mean and variance for *T* are given by

$$E[T]=\frac{1}{\lambda }\frac{\Gamma (k_{1}+\beta^{-1})\Gamma (k_{2}-\beta^{-1})}{\Gamma (k_{1})\Gamma (k_{2})}{\left(\frac{k_{2}}{k_{1}}\right)}^{1/\beta }$$

and

(A11) $$\mathrm{Var}[T]=\frac{1}{\lambda^{2}}\frac{\Gamma (k_{1}+2\beta^{-1})\Gamma (k_{2}-2\beta^{-1})}{\Gamma (k_{1})\Gamma (k_{2})}{\left(\frac{k_{2}}{k_{1}}\right)}^{2/\beta }-E[T{]}^{2}$$

There is no closed form expression for the survivor function, but the *p*th percentile for *T* is given by

*t_p_* = exp(μ)(*w_k_*_1,*k*2,*p*_)^(b/δ)^ (A12)

where *w_k_*_1,*k*2,*p*_ is the pth quantile of an F distribution with (2*k*_1_, 2*k*_2_) degrees of freedom. *t_p_* can thus be computed for various values of *p* and a plot of 1 − *p* against *t_p_* defines the survivor function for *T*.

### Appendix A.1. The Generalized Logistic Family

#### Appendix A.1.1. The Burr XII [46] Distribution (*k*_1_ = 1)

Setting *k*_1_ = 1 into Equation (A3) gives

(A13) $$f(t)=\frac{\left(1/k_{2}\right)\lambda \;\beta }{\left\{\Gamma {(k}_{2})\right\}/\Gamma (1+k_{2})}\frac{{\left(\lambda t\right)}^{\beta \;-1}}{{\left(1+\{{1/k}_{2}\}{\left(\lambda t\right)}^{\beta }\right)}^{\left(1+k_{2}\right)}}$$

Further when *k*_1_ = 1, Г(1 + *k*_2_) = *k*_2_ Г(*k*_2_) and so Г(*k*_2_)/Г(1 + *k*_2_) = 1/*k*_2_ and Equation (A13) becomes

(A14) $$f(t)=\frac{\lambda \;\beta {\left(\lambda t\right)}^{\beta \;-1}}{{\left(1+\{{1/k}_{2}\}{\left(\lambda t\right)}^{\beta }\right)}^{\left(1+k_{2}\right)}}$$

The survivor function is therefore given by

(A15) $$S(t)=1-\int_{0}^{t}f(t)\mathrm{dt}={\left[1+\frac{1}{k_{2}}{\left(\lambda t\right)}^{\beta }\right]}^{-k_{2}}$$

Substituting *k*_1_ = 1 into Equation (A11) leads to the following expressions for the mean and variance of *T*

$$E[T]=\frac{1}{\lambda }\frac{\Gamma (1+\beta^{-1})\Gamma (k_{2}-\beta^{-1})}{\Gamma (k_{2})}{\left(k_{2}\right)}^{1/\beta }$$

and

$$\mathrm{Var}[T]=\frac{1}{\lambda^{2}}\frac{\Gamma (1+2\beta^{-1})\Gamma (k_{2}-2\beta^{-1})}{\Gamma (k_{2})}{\left(k_{2}\right)}^{2/\beta }-E[T{]}^{2}$$

#### Appendix A.1.2. The Burr III Distribution (*k*_2_ = 1)

Setting *k*_2_ = 1 into Equation (A3) gives

(A16) $$f(t)=\frac{k_{1}^{2}\lambda \;\beta {\left(\lambda t\right)}^{k_{1}\beta -1}}{{\left(1+\{1/k_{1}\}{\left(\lambda t\right)}^{\beta }\right)}^{\left(1+k_{1}\right)}}$$

#### Appendix A.1.3. The Log-Logistic [47] Distribution (*k*_1_ = *k*_2_ = 1)

Setting *k*_1_ = *k*_2_ = 1 into Equation (A3) gives

(A17) $$f(t)=\frac{\lambda \;\beta {\left(\lambda t\right)}^{\beta \;-1}}{{\left(1+{\left(\lambda t\right)}^{\beta }\right)}^{2}}$$

The survivor function can easily be found by substituting *k*_2_ = 1 into Equation (A13)

(A18) $$S(t)={\left[1+{\left(\lambda t\right)}^{\beta }\right]}^{-1}$$

Substituting *k*_1_ = *k*_2_ = 1 into Equation (A11) leads to the following expressions for the mean and variance of *T*

$$E[T]=\frac{1}{\lambda }\Gamma (1+\beta^{-1})\Gamma (1-\beta^{-1})$$

and

$$\mathrm{Var}[T]=\frac{1}{\lambda^{2}}\Gamma (1+2\beta^{-1})\Gamma {(k}_{2}-2\beta^{-1})-E[T{]}^{2}$$

The Gumbel [48] distribution has a similar restriction, *k*_1_ = *k*_2_.

#### Appendix A.1.4. The Logistic Distribution (*k*_1_ = *k*_2_ = σ = 1)

σ = 1 implies β = 1, Setting *k*_1_ = *k*_2_ = 1 into Equation (A3) gives

(A19) $$f(t)=\frac{\lambda \;}{1+\lambda t}$$

The survivor function can easily be found by substituting β = 1 into Equation (A17)

(A20) $$S(t)={\left[1+\left(\lambda t\right)\right]}^{-1}$$

### Appendix A.2. The Generalised Gamma Family

#### Appendix A.2.1. The Generalised Gamma Distribution (*k*_2_ = ∞)

Prentice [37] has shown that, when *k*_2_ = ∞, Equation (9c) in Section 3.1.2 reduces to

(A21) $$f(z)=\frac{k_{1}{}^{k_{1}}}{\Gamma (k_{1})}\exp\;\left(k_{1}z-k_{1}e^{z}\right)$$

with $\delta =\sqrt{k_{1}}$ and thus $W=\sqrt{k_{1}}Z=(Y-\mu )\frac{\sqrt{k_{1}}}{b}$. Using the change of variable technique, it follows that

(A22) $$f(y)=f(z)\frac{\partial z}{\partial y}=\frac{1}{b}\frac{k_{1}{}^{k_{1}}}{\Gamma (k_{1})}\exp\;\left(\frac{k_{1}}{b}[y-\mu ]-k_{1}e^{\frac{1}{b}[y-\mu ]}\right)$$

Again, using β = 1/b, λ = exp(−μ) and *T* = exp(*Y*), and the change of variable technique

(A23) $$\begin{array}{cc} f(t) & =f(y)\frac{\partial y}{\delta \;t}=\frac{t^{-1}}{b}\frac{k_{1}{}^{k_{1}}}{\Gamma (k_{1})}\exp\;\;\left(\frac{k_{1}}{b}[y-\mu ]-k_{1}{\left(\lambda t\right)}^{\beta }\right)\\ & =\frac{k_{1}{}^{k_{1}}\lambda \beta }{{\Gamma (k}_{1})}\left({\left(\lambda t\right)}^{k_{1}\beta -1}\exp[-k_{1}{\left(\lambda t\right)}^{\beta }]\right) \end{array}$$

#### Appendix A.2.2. The Weibull (*k*_1_ = 1) [49] and Exponential Distributions (*k*_1_ = 1 = σ)

Inserting *k*_1_ = 1 into Equation (A22) yields the well-known Weibull distribution

(A24) $$f(t)=\frac{\lambda \beta }{\Gamma (1)}\left({\left(\lambda t\right)}^{\beta -1}\exp[-{\left(\lambda t\right)}^{\beta }]\right)=\lambda \beta (\lambda t{)}^{\beta -1}\exp[-{\left(\lambda t\right)}^{\beta }]$$

Closed form expressions exist for the survivor and hazard function of the Weibull

(A25) $$S(t)=1-\int_{0}^{t}f(t)dt=\exp\left[-{\left(\lambda t\right)}^{\beta }\right]\;\mathrm{and}\;h(t)\;=\;-\frac{\mathrm{dln}[S(t)]}{dt}=\beta \lambda {\left(\lambda t\right)}^{\beta -1}$$

As *k*_2_ tends to infinity it can be shown that

(A26) $$\frac{\Gamma (k_{2}-\sigma )}{\Gamma (k_{2})}{\left(k_{2}\right)}^{\sigma }\to \;1\;\mathrm{and}\;\frac{\Gamma (k_{2}-2\sigma )}{\Gamma (k_{2})}{\left(k_{2}\right)}^{2\sigma }\to \;1\;$$

and substituting this expression and *k*_1_ = 1 into Equation (A11) gives the formulas for the mean and variance of *T* when this variable is Weibull distributed

(A27) $$E[T]=\frac{e^{\mu }\Gamma (k_{1}+\sigma )\Gamma (k_{2}-\sigma )}{\Gamma (k_{1})\Gamma (k_{2})}\left(\frac{k_{2}}{k_{1}}\right)=e^{\mu }\Gamma (1+\sigma )=\frac{1}{\lambda }\Gamma (1+1/\beta )$$

(A28) $$\begin{array}{cc} \mathrm{Var}[T] & =\frac{e^{2\mu }\Gamma (k_{1}+2\sigma )\Gamma (k_{2}-2\sigma )}{{\Gamma (k}_{1})\Gamma {(k}_{2})}{\left(\frac{k_{1}}{k_{2}}\right)}^{2\sigma }-E[T{]}^{2}=e^{2\mu }\Gamma (1+2\sigma )-E[T{]}^{2}\\ & =\frac{1}{\lambda^{2}}\Gamma (1+2/\beta )-\frac{1}{\lambda^{2}}\Gamma^{2}(1+1/\beta ) \end{array}$$

If σ also equals 1, then β = 1 and the Weibull distribution collapses to the exponential distribution

(A29) $$f(t)=\lambda (\lambda t{)}^{1-1}\exp[-{\left(\lambda t\right)}^{1}]=\lambda \exp[-(\lambda t)]$$

Substituting β = 1 into the survivor and hazard functions of the Weibull distribution then gives these functions for the exponential distribution

(A30) $$S(t)=1-\int_{0}^{t}f(t)dt=\exp(-\lambda t)\;\mathrm{and}\;h(t)\;=-\frac{\mathrm{dln}[S(t)]}{\mathrm{dt}}=\lambda$$

Given Г(2) = Г(1) = 1 and, Г(3) = 2,

(A31) $$E[T]=\frac{1}{\lambda }\Gamma (1+1)=\frac{1}{\lambda }\;\mathrm{and}\;\mathrm{Var}[T]=\frac{1}{\lambda^{2}}\Gamma (1+2)-\frac{1}{\lambda^{2}}\Gamma^{2}(1+1)=\frac{2}{\lambda^{2}}-\frac{1}{\lambda^{2}}=\frac{1}{\lambda^{2}}$$

#### Appendix A.2.3. The Gamma [50] Distribution (σ = 1).

σ = 1 implies β = $1/\sqrt{k_{1}}$ and so

(A32) $$f(t)=\frac{\sqrt{k_{1}}\;\lambda }{\Gamma (k_{1})}\left({\left(\lambda t\right)}^{{\sqrt{k}}_{1}-1}\exp[k_{1}{\left(\lambda t\right)}^{\sqrt{k_{1}}}]\right)$$

#### Appendix A.2.4. The Log Normal Distribution (*k*_1_ = *k*_2_ = ∞)

Lawless [51] has shown that the distribution for *W* when *k*_2_ = ∞

(A33) $$f(w)=\frac{k_{1}{}^{k_{1}-0.5}}{\Gamma (k_{1})}\exp\;\left(\sqrt{k_{1}}w-k_{1}e^{w/\sqrt{k_{1}}}\right)$$

tends to

(A34) $$f(w)=\frac{1}{\sqrt{2\pi \sigma^{2}}}\exp\;\left(-0.5w^{2}\right)$$

as *k*_1_ → ∞. This is the equation for a standard normal distribution, so *W* is a standard normal variate with mean zero and standard deviation of 1. This then implies *Y* is normally distributed and therefore *T* is log normally distributed with pdf

(A35) $$f(t)=\frac{1}{\sigma \;t\sqrt{2\pi }}\exp\;\left(-0.5\frac{{\left(\ln(t)-\mu \right)}^{2}}{\sigma^{2}}\right)$$
